# Prenatal and early childhood exposure to tetrachloroethylene (PCE)-contaminated drinking water and sleep quality in adulthood: a retrospective cohort study

**DOI:** 10.1186/s12940-021-00819-7

**Published:** 2022-01-15

**Authors:** Charlotte R. Doran, Ann Aschengrau

**Affiliations:** Department of Epidemiology, Boston University School of Public Health, 715 Albany Street, Talbot 328 east, Boston, MA 02118 USA

## Abstract

**Background:**

Communities in Cape Cod, Massachusetts were exposed to tetrachloroethylene (PCE) through contaminated drinking water from 1969 to 1983. PCE exposure during adulthood has well-established neurotoxic effects; however, long-term impacts stemming from early life exposure, especially adverse effects on sleep quality, are not well understood.

**Methods:**

The present analysis was based on data from the Cape Cod Health Study, a retrospective cohort study of the long-term neurotoxic impacts of early-life exposure to PCE-contaminated drinking water. Exposure to PCE-contaminated water was estimated using a validated leaching and transport model. Measures of sleep quality were obtained from self-administered questionnaires. Generalized estimating equations were used to generate risk ratios and 95% confidence intervals to estimate the association between early-life PCE exposure and sleep quality among 604 participants.

**Results:**

Compared to unexposed participants, any PCE exposure during early life was associated with 1.57 times the risk of reporting breathing pauses during sleep (95% CI 0.92–2.68). Low-level exposure to PCE was associated with 1.50 times the risk of reporting sleep apnea or other sleep disorders (95% CI 0.78–2.89), while high levels of exposure had comparable risk compared to no exposure (RR = 0.94, 95% CI 0.50–1.79). Weak or no associations were observed for other sleep quality outcomes. In stratified analyses participants with mental illness and/or substance use disorder had increased risk ratios for short sleep duration associated with PCE exposure.

**Conclusion:**

These findings suggest that early-life exposure to PCE may be associated with a moderate increase in the risk of reporting breathing pauses during sleep in adulthood and that a history of mental illness and/or substance use disorder may exacerbate the risk of short sleep duration.

## Introduction

Tetrachloroethylene, also known as perchloroethylene (PCE), is a common organic solvent most often used in dry cleaning and metal degreasing. Workers in these industries are often exposed to high levels of PCE [1]. Exposure to PCE and other organic solvents in occupational settings have been shown to have neurotoxic effects, including adverse effects on color vision, visuospatial memory, cognition, and other neuropsychological functions [1–3]. In the past two decades, there has been increasing interest in the long-term neurotoxic effects of PCE exposure, including the effects of early-life PCE exposure. In 2001, Till et al. showed in a prospective cohort study of the offspring of organic solvent-exposed and unexposed women that maternal exposure to these solvents was associated with an increased risk of color vision and visual acuity impairment in offspring [4]. While industrial use of PCE is a key source of high exposure levels in occupational settings, community exposures through contaminated drinking water and air are important sources of lower but more widespread exposure levels, and little is known about the health effects of PCE exposure in the community setting [1].

Between the years 1968 and 1980, contamination of public drinking water with PCE occurred in some neighborhoods of Cape Cod, Massachusetts via the installation of vinyl-lined (VL) asbestos cement (AC) water pipes [5]. The lining was composed of vinyl dissolved in PCE which, since PCE is volatile, was expected to evaporate before the pipes were installed. However, water samples taken in 1980 showed that substantial amounts of PCE remained in the lining and were leaching into the public water supply. In order to understand the long-term health effects of prenatal and early childhood PCE exposure in the affected communities, the Cape Cod Health Study (CCHS) recruited individuals who were born and lived on Cape Cod during the period in which drinking water was contaminated. The CCHS has shown that exposure to PCE among these individuals in early life is associated with neurotoxic effects such as long-term color vision dysfunction [6], in addition to adverse behavioral and mental health outcomes such as increased risk of drug use disorder, bipolar disorder, and post-traumatic stress disorder [5, 7, 8].

Though understanding of long-term effects of PCE exposure is growing, few studies at present have been conducted to examine the effects of prenatal and early childhood PCE exposure on sleep disorders and quality of sleep in adulthood. Occupational exposure to organic solvents can have adverse effects on sleep quality [9] and long-term exposure may be associated with an increased risk of sleep apnea [10], although these findings are inconsistent across studies [11]. Symptoms of sleep apnea include snoring, breathing pauses during sleep, difficulty staying asleep, and difficulty staying awake. In some cases, symptoms can also include gasping for air during sleep and excessive daytime sleepiness. It is estimated that obstructive sleep apnea affected up to 38% of the general adult population as of 2017 [12], with over 80% of cases being undiagnosed [13]. The estimated prevalence of sleep apnea is high in comparison to other sleep disorders such as restless legs syndrome (RLS), which has an estimated prevalence in adults of less than 15% [14] and narcolepsy, which is rare and affects approximately 1 in 2000 adults [15]. Sleep difficulties have been linked to deficiencies in attention, visual memory, and broader cognitive function [16]. Additionally, inadequate sleep duration has been shown to be associated with severe psychological distress, depressive symptoms, and perceived stress severity among adults [17, 18]. Adults who have a short sleep duration are also more likely to report having a chronic health condition compared to those who get adequate sleep [19].

This study aims to continue the exploration of the psychological and physiological effects of prenatal and early childhood PCE exposure by evaluating its association with several sleep quality outcomes.

## Methods

### Study population

Individuals were eligible for inclusion in the CCHS if they were born during 1969–1983 to married women living in any of eight Cape Cod, Massachusetts towns known to have some VL/AC water pipes installed in some parts of their water distribution system. The towns included were Barnstable, Bourne, Falmouth, Mashpee, Sandwich, Brewster, Chatham, and Provincetown. Potentially eligible individuals were identified by reviewing birth certificates and crossmatching the maternal address on the certificate with information collected from water companies on the location, installation year, and diameter of all VL/AC pipes in the water distribution system. Initial tentative exposure assignments were made by visual inspection of water distribution maps as described by Aschengrau et al. [7]. In total, 1910 potentially exposed subjects and 1928 frequency-matched (by birth month and year) potentially unexposed subjects were identified for recruitment. 1202 older siblings were also identified for recruitment. Individuals for whom a current address was found were recruited by mail. Persistent non-responders were contacted by telephone if a phone number was found. Final exposure status used in analysis was determined using a leaching and transport model developed by Webler and Brown [20]. The model estimates the amount of PCE delivered to the household by using the initial amount of PCE in the pipe lining, the age of the pipe, and the leaching rate of PCE from the vinyl lining into the water.

**Fig. 1:**
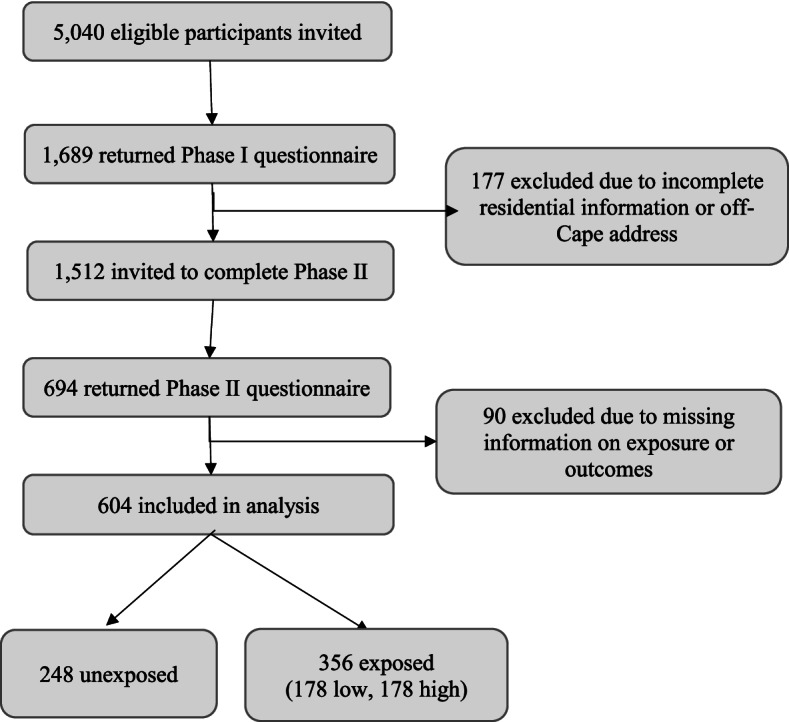
Selection and enrollment of participants in the Cape Cod Health Study and inclusion in analysis sample

Participants were sent two surveys, with the Phase I survey sent between 2000 and 2005, and the Phase II survey between 2017 and 2020. Each survey collected data on demographics, health conditions, mental health, and behaviors. The Phase II survey also included questions on sleep quality. In total, 1689 participants returned the Phase I questionnaire, of whom 1512 were eligible for Phase II. 694 of these eligible participants completed the Phase II questionnaire. 90 participants were excluded from the current analysis due to incomplete information on PCE exposure or sleep quality outcomes. Thus, the final analysis population included 604 participants. Figure 1 shows a flow chart of participant selection, enrollment, and inclusion in the current analysis.

### Exposure and outcome assessment

PCE exposure was first defined as a binary exposure variable in which participants were categorized by any or no pre- and post-natal PCE exposure as determined by the leaching and transport model. There were 248 participants with no PCE exposure, and 356 participants with any PCE exposure. The minimum level of PCE exposure among exposed participants was 0.04 g and the maximum was 3722.22 g with a median of 35.51 g (interquartile range 7.55 g to 122.73 g).

PCE exposure was then classified into a three-level ordinal exposure variable of unexposed, low exposure, and high exposure. Unexposed individuals were those who had no pre- or postnatal PCE exposure (*N* = 248). Exposed individuals were classified into high (*N* = 178) and low exposure groups (N = 178) by a median exposure cutoff. The median PCE exposure level was determined based on the distribution of PCE exposure levels in the remaining 356 exposed participants. Demographic characteristics, health history, parental and family history, and pre- and peri-natal characteristics were evaluated by the two-level exposure status.

Reported sleep outcomes examined were: diagnosis of sleep apnea or other sleep disorder, average sleep duration per 24-h period, difficulty falling asleep, feeling poorly rested, difficulty staying awake, breathing pauses during sleep, and snoring. Sleep duration, difficulty falling asleep, feeling poorly rested, and difficulty staying awake were evaluated during the 30 days prior to survey completion. All other questions referred to any past occurrence. Participants who responded that their average sleep duration was less than 7 h per 24-h period were considered to have short sleep duration [21]. For questions that were asked using a 4-level Likert scale, dichotomous outcomes were derived for use in analysis by mapping responses of “Often” and “Sometimes” to indicate affirmative and “Rarely” and “Never” to indicate negative. Finally, a composite sleep outcome variable was derived to indicate the number of poor sleep indicators answered in the affirmative, ranging from 0, meaning absence of any poor sleep indicators, to 7. Due to limitations of model convergence and the small number of participants with 0 or 1 poor sleep quality indicators, the overall number of sleep quality indicators was analyzed as a dichotomous outcome comparing the risk of 3 or more poor sleep quality indicators against 0–2 in order to provide sufficient subjects in analysis categories. Other analyses not shown indicate that different ways to operationalize this variable did not impact the conclusions.

### Analysis

All analyses were conducted using SAS Version 9.4. Risk ratios were used to estimate the magnitude of the association between PCE exposure and sleep outcomes. 95% confidence intervals were generated around the point estimates to assess their precision. The presence of outliers was assessed for duration of sleep, as the only continuous outcome assessed. Values were considered outliers if the response was greater than 24 h in a 24-h period. No outliers were identified in the analysis dataset. Due to the inclusion of siblings of participants in the study population, observations within families were assumed to be non-independent. As such, generalized estimating equation (GEE) analyses using the logit link were used to account for correlation within families.

Finally, adjusted GEE analyses were conducted to control for confounding variables. While many covariates collected are considered risk factors for poor sleep indicators, potential confounders were considered for inclusion in multivariate models only if they had a plausible causal relationship with both prenatal and early childhood exposure to PCE and sleep outcomes. Covariates that met these criteria were the participant’s age, mother’s education level, and father’s occupation. These covariates were selected into the final analysis by forward stepwise selection with a 10% change-in-estimate approach. Confounders that were identified for any of the sleep outcomes were included in all sleep outcome analyses. Following the forward stepwise selection process, age and father’s occupation were determined to be confounders and included in the models. Finally, stratified analyses were conducted by reported history of mental illness and substance use disorder (including alcohol and drug use disorder), and marital status.

## Results

Of 604 participants included in the analysis, 248 participants were unexposed and 356 participants were exposed. Table 1 shows the distribution of selected characteristics by PCE exposure status. The characteristics of both groups were similar. Slightly over half of the participants were born between 1975 and 1980, and the mean age between groups was similar. The participants were predominantly white, female, and college graduates, and the majority were employed and either married or cohabitating at the time of survey completion. Few participants had possible occupational exposure to solvents, but many had potential exposure through hobbies. A minority of participants had mothers with potential occupational exposure to solvents. Most mothers did not smoke cigarettes or marijuana during gestation, and about half of the mothers did not drink during gestation. Demographic and health characteristics were examined by 3-level exposure status in analyses not shown, and all exposure groups were similar with respect to these characteristics.

**Table 1:** Distribution of baseline characteristics by exposure status among CCHS^a^ Phase II analysis population, 2017–2020

	Prenatal and Early Childhood PCE Exposure Status^b^	
	Unexposed	Exposed
Characteristic	N = 248	***N*** = 356
**Demographics**		
Current Age, mean ± SD	40.7 ± 3.8	40.3 ± 3.7
Year of birth, n (%)		
1969–1974	64 (25.8%)	76 (21.4%)
1975–1980	130 (52.4%)	186 (52.3%)
1981–1983	54 (21.8%)	94 (26.4%)
Race, n (%)		
White	246 (99.2%)	352 (98.9%)
Non-white	2 (0.8%)	4 (1.1%)
Sex at birth, n (%)		
Female	173 (69.8%)	224 (62.9%)
Male	75 (30.2%)	132 (37.1%)
Educational level, n (%)		
High school graduate or less	15 (6.1%)	18 (5.1%)
Some college or 2-year degree	45 (18.2%)	61 (17.2%)
4-year college grad or higher	187 (75.7%)	276 (77.8%)
Missing	1	1
Currently employed, n (%)		
Yes	219 (90.1%)	320 (90.4%)
No	24 (9.9%)	34 (9.6%)
Missing	5	2
Current marital status, n (%)		
Married or cohabitating	197 (79.4%)	308 (86.5%)
Separated	3 (1.2%)	1 (0.3%)
Divorced	21 (8.5%)	16 (4.5%)
Widowed	0 (0.0%)	1 (0.3%)
Never Married	27 (10.9%)	30 (8.4%)
**Behavioral and Health History**		
Ever had solvent-exposed job, n (%)		
Yes	34 (14.1%)	54 (15.6%)
No	208 (86.0%)	292 (84.4%)
Missing	6	10
Ever had solvent-exposed hobby, n (%)		
Yes	216 (87.8%)	299 (84.7%)
No	30 (12.2%)	54 (15.3%)
Missing	2	3
History of learning problem, n (%)		
Yes	43 (17.5%)	64 (18.3%)
No	203 (82.5%)	286 (81.7%)
Missing	2	6
Ever repeated grade, n (%)		
Yes	27 (10.9%)	33 (9.4%)
No	220 (89.1%)	318 (90.6%)
Missing	1	5
History of military service, n (%)		
Yes	16 (6.5%)	20 (5.6%)
No	232 (93.6%)	336 (94.4%)
History of mental disorder, n (%)		
Yes	91 (36.8%)	146 (41.4%)
No	156 (63.2%)	207 (58.6%)
Missing	1	3
**Family Characteristics**		
Mother’s age at subject’s birth (years), mean ± SD	27.7 ± 4.4	27.5 ± 4.5
Father’s age at subject’s birth (years), mean ± SD	30.0 ± 5.3	29.9 ± 5.5
Mother’s educational level at subject’s birth, n (%)		
High school graduate or less	69 (27.9%)	119 (33.4%)
Some college	88 (35.6%)	101 (28.4%)
4-year college graduate or higher	90 (36.4%)	136 (38.2%)
Missing	1	0
Mother’s occupational exposure to solvents, n (%)		
Yes	26 (13.1%)	45 (15.0%)
No	173 (86.9%)	256 (85.1%)
Missing	49	55
Father’s occupation at subject’s birth, n (%)		
White collar	121 (49.2%)	204 (57.8%)
Blue collar	76 (30.9%)	105 (29.8%)
Other	49 (19.9%)	44 (12.5%)
Missing	2	3
Number of older siblings, n (%)		
0	116 (47.2%)	167 (46.9%)
1	78 (31.7%)	124 (34.8%)
2+	52 (21.1%)	65 (18.3%)
Missing	2	0
Family history of alcohol problem, n (%)		
Yes	103 (41.7%)	170 (47.8%)
No	136 (55.1%)	177 (49.7%)
Don’t know	8 (3.2%)	9 (2.5%)
Missing	1	0
Family history of drug problem, n (%)		
Yes	54 (21.8%)	80 (22.5%)
No	181 (73.0%)	257 (72.2%)
Don’t know	13 (5.2%)	19 (5.3%)
**Perinatal Characteristics**		
Subject’s birth weight (g), mean ± SD	3398.8 ± 556.4	3425.9 ± 506.9
Subject’s gestational age (months), mean ± SD	39.9 ± 2.2	40.1 ± 2.6
Mother received prenatal care during subject’s gestation, n (%)		
Yes	236 (100.0%)	345 (99.7%)
No	0 (0.0%)	1 (0.3%)
Missing	12	10
Mother’s cigarette smoking during subject’s gestation, n (%)		
Didn’t smoke	158 (77.8%)	233 (76.6%)
1–10 cigarettes/day	22 (10.8%)	32 (10.5%)
11+ cigarettes/day	23 (11.3%)	39 (12.8%)
Missing	45	52
Mother’s alcohol consumption during subject’s gestation, n (%)		
Didn’t drink	115 (56.7%)	155 (51.2%)
1–3 drinks/month	60 (29.6%)	85 (28.1%)
1+ drinks/week	28 (13.8%)	63 (20.8%)
Missing	45	53
Mother’s use of marijuana during subject’s gestation, n (%)		
Yes	9 (4.5%)	10 (3.3%)
No	192 (95.5%)	295 (96.7%)
Missing	47	51
Mother’s medical and obstetrical complications during subject’s gestation, n (%)		
Yes	50 (25.0%)	59 (19.5%)
No	150 (75.0%)	243 (80.5%)
Missing	48	54
Multiple pregnancy, n (%)		
Yes	10 (4.0%)	8 (2.3%)
No	238 (96.0%)	348 (97.8%)
Subject breast fed, n (%)		
Yes	137 (68.5%)	198 (65.6%)
No	63 (31.5%)	104 (34.4%)
Missing	48	54

^a^ Cape Cod Health Study

^b^ Participants are considered exposed if they have any pre- or postnatal PCE exposure, and unexposed if they have no exposure

^c^ All percentages are presented as proportions of non-missing information

569 participants (94.2%) reported at least one poor sleep quality outcome. The most commonly reported poor sleep outcomes were trouble staying awake (*N* = 433, 71.7%) and difficulty falling asleep (*N* = 306, 50.7%). Difficulty falling asleep, trouble staying awake, and feeling poorly rested were often reported together. Breathing pauses were commonly reported among participants who also reported being diagnosed with sleep apnea or another sleep disorder (*n* = 21, 42.0%). Among participants who reported breathing pauses during sleep (*N* = 56), almost all (*N* = 45, 80.4%) also reported snoring.

Table 2 shows the crude and adjusted GEE risk ratios for any early-life exposure to PCE and seven sleep outcomes, including diagnosis of sleep apnea or other sleep disorder, average sleep duration per 24-h period, difficulty falling asleep, feeling poorly rested, difficulty staying awake, breathing pauses during sleep, and snoring. Participants exposed to PCE had a 57% increased risk of reporting breathing pauses during sleep (adjusted RR = 1.57 [0.92, 2.68]) compared to unexposed participants. A slight 19% decreased risk in feeling poorly rested was observed among PCE-exposed participants (adjusted RR = 0.81 [0.64, 1.03]). Weak associations were observed on the effect of PCE exposure on reporting sleep apnea or other sleeping disorder (adjusted RR = 1.17 [0.69, 1.99]), having a short sleep duration (adjusted RR = 1.14 [0.86, 1.51]), and snoring (adjusted RR = 1.16 [0.88, 1.52]). Additionally, there were no associations or weak associations observed with difficulty falling asleep (adjusted RR = 0.93 [0.79, 1.09]), trouble staying awake (adjusted RR = 1.00 [0.90, 1.10]), and the overall number of poor sleep quality indicators (adjusted RR = 0.94 [0.77, 1.13]).

**Table 2:** Crude and adjusted risk ratios (RR) and 95% confidence intervals (CI) between PCE exposure status and quality of sleep, CCHS^a^ 2017–2020

	Outcome Status		Crude GEE Analysis^**b**^	Adjusted GEE Analysis^**c**^
Outcome			RR (95% CI)^d^	RR (95% CI)
**Sleep apnea/Other sleep disorder**	**Yes**	**No**		
Unexposed	20	228	1.00 (ref)	1.00 (ref)
Exposed	30	326	1.05 (0.62, 1.78)	1.17 (0.69, 1.99)
**Breathing pauses**	**Yes**	**No**		
Unexposed	19	229	1.00 (ref)	1.00 (ref)
Exposed	37	319	1.36 (0.80, 2.30)	1.57 (0.92, 2.68)
**Snoring**	**Yes**	**No**		
Unexposed	63	185	1.00 (ref)	1.00 (ref)
Exposed	100	256	1.11 (0.84, 1.45)	1.16 (0.88, 1.52)
**Short sleep duration**	**< 7 h**	**7 h or more**		
Unexposed	59	189	1.00 (ref)	1.00 (ref)
Exposed	97	259	1.14 (0.87, 1.51)	1.14 (0.86, 1.51)
**Difficulty falling asleep**	**Sometimes/Often**	**Rarely/Never**		
Unexposed	131	117	1.00 (ref)	1.00 (ref)
Exposed	175	181	0.93 (0.79, 1.10)	0.93 (0.79, 1.09)
**Poorly rested**	**Sometimes/Often**	**Rarely/Never**		
Unexposed	89	159	1.00 (ref)	1.00 (ref)
Exposed	103	253	0.81 (0.64, 1.02)	0.81 (0.64, 1.03)
**Trouble staying awake**	**Sometimes/Often**	**Rarely/Never**		
Unexposed	178	70	1.00 (ref)	1.00 (ref)
Exposed	255	101	1.00 (0.91, 1.10)	1.00 (0.90, 1.10)
**Number of poor sleep quality indicators**	**3 or more**	**Less than 3**		
Unexposed	112	136	1.00 (ref)	1.00 (ref)
Exposed	149	207	0.92 (0.76, 1.11)	0.94 (0.77, 1.13)

^a^ CCHS = Cape Cod Health Study

^b^ The crude GEE analysis and adjusted GEE analysis are conducted accounting for correlations within families

^c^ Adjusted for age and father's occupational status

^d^ RR = Risk Ratio

Table 3 shows the crude and adjusted GEE risk ratios for sleep outcomes using the three-level ordinal PCE exposure variable. In general, the magnitude of association was highest in the low exposure group, then decreased in the high exposure group relative to the unexposed reference group. Low levels of PCE exposure was associated with a 50% increased risk of being diagnosed with sleep apnea or another sleep disorder compared to no exposure (adjusted RR = 1.50 [0.78,2.89]). However, a positive association was not present among highly exposed participants (adjusted RR = 0.94 [0.50, 1.79]). Similarly, low exposure was associated with an 81% increased risk of experiencing breathing pauses during sleep (adjusted RR = 1.81 [0.97, 3.38]), and high exposure was associated with a lower 33% increased risk (adjusted RR = 1.33 [0.70, 2.52]) compared to no exposure. There were weak associations between PCE and snoring among both the low exposure and high exposure groups compared to the unexposed group (adjusted RR = 1.14 [0.82, 1.59] and adjusted RR = 1.17 [0.86, 1.61], respectively).

**Table 3:** Crude and adjusted risk ratios (RR) and 95% confidence intervals (CI) between PCE exposure level and quality of sleep, CCHS^a^ 2017–2020

	Outcome status		Crude GEE Analysis^b^	Adjusted GEE Analysis^c^
Outcome			RR^d^ (95% CI)	RR (95% CI)
**Sleep apnea/other sleep disorder**	**Yes**	**No**		
Unexposed	20	228	1.00 (ref)	1.00 (ref)
Low exposure	16	162	1.13 (0.61, 2.10)	1.50 (0.78, 2.89)
High exposure	14	164	0.97 (0.51, 1.82)	0.94 (0.50, 1.79)
**Breathing pauses**	**Yes**	**No**		
Unexposed	19	229	1.00 (ref)	1.00 (ref)
Low exposure	21	157	1.54 (0.86, 2.77)	1.81 (0.97, 3.38)
High exposure	16	162	1.17 (0.62, 2.22)	1.33 (0.70, 2.52)
**Snoring**	**Yes**	**No**		
Unexposed	51	127	1.00 (ref)	1.00 (ref)
Low exposure	49	129	1.09 (0.79, 1.51)	1.14 (0.82, 1.59)
High exposure	63	185	1.12 (0.81, 1.53)	1.17 (0.86, 1.61)
**Short sleep duration**	**< 7 h**	**≥7 h**		
Unexposed	59	189	1.00 (ref)	1.00 (ref)
Low exposure	51	127	1.21 (0.89, 1.67)	1.29 (0.94, 1.78)
High exposure	46	132	1.07 (0.77, 1.50)	1.00 (0.70, 1.42)
**Difficulty falling asleep**	**Sometimes/Often**	**Rarely/Never**		
Unexposed	131	117	1.00 (ref)	1.00 (ref)
Low exposure	87	91	0.93 (0.76, 1.12)	0.91 (0.75, 1.12)
High exposure	88	90	0.94 (0.77, 1.14)	0.94 (0.77, 1.15)
**Feeling poorly rested**	**Sometimes/Often**	**Rarely/Never**		
Unexposed	89	159	1.00 (ref)	1.00 (ref)
Low exposure	48	130	0.75 (0.56, 1.01)	0.79 (0.58, 1.07)
High exposure	55	123	0.86 (0.66, 1.14)	0.83 (0.62, 1.10)
**Trouble staying awake**	**Sometimes/Often**	**Rarely/Never**		
Unexposed	178	70	1.00 (ref)	1.00 (ref)
Low exposure	123	55	0.97 (0.85, 1.10)	0.95 (0.83, 1.08)
High exposure	132	46	1.03 (0.92, 1.16)	1.05 (0.93, 1.18)
**Number of poor sleep quality indicators**	**3 or more**	**Less than 3**		
Unexposed	112	136	1.00 (ref)	1.00 (ref)
Low exposure	70	108	0.86 (0.68, 1.09)	0.90 (0.71, 1.14)
High exposure	79	99	0.98 (0.79, 1.22)	0.97 (0.77, 1.22)

^a^ CCHS = Cape Cod Health Study

^b^ The crude GEE analysis and adjusted GEE analysis are conducted accounting for correlations within families

^c^ Adjusted for father’s occupation at subject’s birth and subject age

^d^ RR = Risk Ratio

Low PCE exposure was associated with a 29% increased risk of short sleep duration compared to no exposure (adjusted RR = 1.29 [0.94, 1.78]), but high exposure had no observed difference in risk (adjusted RR = 1.00 [0.70, 1.42]). PCE exposure was associated with similar decreased risks of feeling poorly rested in both the low and high exposure groups (adjusted RR = 0.79 [0.58, 1.07] for low exposure, adjusted RR = 0.83 [0.62, 1.10] for high exposure). No or weak associations were observed with difficulty falling asleep (adjusted RR_low_ = 0.91 [0.75, 1.12], adjusted RR_high_ = 0.94 [0.77, 1.15]), trouble staying awake (adjusted RR_low_ = 0.95 [0.83, 1.08], adjusted RR_high_ = 1.05 [0.93, 1.18]), and the number of poor sleep quality indicators (adjusted RR_low_ = 0.90 [0.71, 1.14] and adjusted RR_high_ = 0.97 [0.77, 1.22]).

Stratified adjusted GEE analyses of 2-level PCE exposure showed that, among participants with mental illness, PCE exposure was associated with a 55% higher risk of short sleep duration (adjusted RR = 1.55, 95% CI 1.00 to 2.38) compared to PCE unexposed, whereas there was no positive association observed among those without mental illness (adjusted RR = 0.89, 95% CI 0.62 to 1.29). A similar modification of effect was observed after stratifying by a history of substance use disorder in which PCE exposure was associated with a 45% higher risk of short sleep duration (adjusted RR = 1.45, 95% CI 1.01 to 2.09) among participants with a positive history of substance use disorder, whereas a null association was observed between PCE exposure and short sleep duration among participants without a positive history (adjusted RR = 0.99, 95% CI 0.60 to 1.65). Participants with substance use disorder had a reduced impact of PCE exposure on feeling poorly rested (RR = 0.62, 95% CI 0.46 to 0.84), while participants without substance use disorder showed a small increase in risk of feeling poorly rested associated with PCE exposure (RR = 1.25, 95% CI 0.83 to 1.87).

## Discussion

These results suggest that prenatal and early childhood exposure to PCE through drinking water contamination may have adverse effects on sleep quality and contribute to the development of sleep disorders in adulthood. Moderate increases in the risk of breathing pauses during sleep were observed in PCE-exposed individuals compared to unexposed individuals. Interestingly, this association did not follow a traditional dose-response relationship. Instead, the largest magnitude of association was observed among the low exposure group, and the association was attenuated or null when comparing the high exposure group to the unexposed group. This pattern of association, which is likely due to bidirectional misclassification of the exposure, in which participants with high exposure were categorized as low exposure, and vice versa, was observed in a previous study of drug-use disorder following early life exposure to PCE-contaminated drinking water conducted in the CCHS population which used a similar median-cutoff definition of exposure levels [8].

We observed a moderate positive association between PCE exposure and short sleep duration among participants with a history of mental illness and among participants with a history of substance use disorder. No associations were observed among participants without mental illness or substance use disorder or among the study population as a whole. PCE exposure has previously been shown to be associated with some mental illness diagnoses and with drug use disorder in the CCHS cohort [5, 7, 8]. The current finding may be due to an interaction between PCE exposure and mental illness or substance use disorder. It is also possible that these conditions are mediators of the relationship between PCE exposure and short sleep duration, in which case the stratified risk ratios are difficult to interpret, as mental illness has been shown to be associated with abnormal sleep duration [22].

The findings presented in this paper should be interpreted in light of the study limitations. First, it was not possible to directly measure PCE exposure among participants during the time period of interest. Therefore, this study used a leaching and transport model to estimate the magnitude of PCE exposure delivered to the home. This model assumes that the conditions of the water delivery system in 1980 were representative of the conditions during the entire exposure assessment period. This assumption was regarded as reasonable given that there were minimal changes to the system during that period. In addition, given the modeled exposure represented the annual mass of PCE delivered to the participant’s residence during gestation and early childhood, the analyses assume similar patterns of exposure to contaminated water in each household, and does not take into account differences in bathing practices and water consumption due to poor recall of these behaviors. A previous study in PCE exposure and breast cancer found that these behaviors were not highly variable and therefore did not impact the exposure assessment [23].

Second, this study uses self-reported measures of sleep quality which may be subject to misclassification bias. Because PCE exposure status was assessed independently from the administration of the CCHS questionnaires, we think that the outcome misclassification is likely nondifferential, and therefore biased the results of the analysis of three-level exposure towards the null. Diagnoses or indications by a healthcare provider of sleep apnea or other sleep disorders were also self-reported. It is likely that these diagnoses are underreported, as it is estimated that more than 80% of cases of sleep apnea are undiagnosed [13].

Another limitation of this study is that diagnoses of sleep apnea and other sleep disorders are indistinguishable in the CCHS Phase II data because they come from a single questionnaire item. As such, direct conclusions regarding the association between PCE exposure and diagnosed sleep apnea alone are not possible, though sleep apnea is common relative to other diagnosable sleep disorders. However, due to the high rate of undiagnosed sleep apnea, an analysis of diagnosed sleep apnea may not be able to provide a good estimate of association. This study found that PCE exposure was associated with an increase in the risk of breathing pauses during sleep, which is a hallmark symptom of sleep apnea. Future studies may consider evaluating sleep apnea and other sleep disorders such as RLS and narcolepsy separately, as well as their associated symptoms, in order to better characterize the association.

A further limitation is the low response rate seen in the Phase II questionnaire. Of the 1512 participants invited to complete Phase II, 694 participants returned the questionnaire, representing an attrition of 54%. However, analysis of available characteristics between the Phase I and Phase II population shows that these participants were similar, and the distribution of characteristics were also balanced between exposure groups. A larger sample size is needed to increase the precision of the effect estimates examined in this study.

While its small size and lipid solubility allows PCE to easily cross the placenta and blood brain barrier and selectively concentrate in the brain and other lipophilic tissue [1], the biological mechanism by which early life exposure to PCE and related solvents might contribute to poor sleep quality later in life is currently unknown. However, available evidence suggests that PCE and related compounds may exert neurotoxic actions on the brain networks involved in sleep and wakefulness via the peroxidation of cell membrane lipids [24], changes in the fatty acids [25], demyelination of nerve cells [26], and changes in GABA_A_ receptors [27].

## Conclusions

The results of this study suggest that PCE exposure in early childhood may have adverse effects on sleep quality and contribute to the development of sleep disorders in adulthood, particularly the risk of experiencing breathing pauses during sleep, a hallmark symptom of sleep apnea. “Natural experiments” in human populations such as the CCHS provide an opportunity to understand the long-term health implications of early-life PCE exposure in community settings.

## Data Availability

The datasets generated and analyzed during this study are not publicly available due to IRB restrictions. Non-identifiable data are however available from the authors upon reasonable request and with permission from the IRBs at Boston University and the Massachusetts Department of Public Health.
